# The mechanism of the molecular CISS effect in chiral nano-junctions

**DOI:** 10.1039/d4sc04435e

**Published:** 2024-08-16

**Authors:** Thi Ngoc Ha Nguyen, Georgeta Salvan, Olav Hellwig, Yossi Paltiel, Lech Thomasz Baczewski, Christoph Tegenkamp

**Affiliations:** a Solid Surface Analysis, Institute of Physics, Chemnitz University of Technology 09126 Chemnitz Germany christoph.tegenkamp@physik.tu-chemnitz.de; b Semiconductor Physics, Institute of Physics, Chemnitz University of Technology 09126 Chemnitz Germany; c Functional Magnetic Materials, Institute of Physics, Chemnitz University of Technology 09126 Chemnitz Germany; d Institute of Ion Beam Physics and Materials Research, Helmholtz-Zentrum Dresden-Rossendorf 01328 Dresden Germany; e Department of Applied Physics, Hebrew University of Jerusalem 91904 Jerusalem Israel; f Center for Nanoscience and Nanotechnology, Hebrew University of Jerusalem 91904 Jerusalem Israel; g Institute of Physics, Polish Academy of Sciences 02-668 Warszawa Poland

## Abstract

The chirality induced spin selectivity (CISS) effect has been up to now measured in a wide variety of systems but its exact mechanism is still under debate. Whether the spin polarization occurs at an interface layer or builds up in the helical molecule is yet not clear. Here we have investigated the current transmission through helical polyalanine molecules as a part of a tunnel junction realized with a scanning tunneling microscope. Depending on whether the molecules were chemisorbed directly on the magnetic Au/Co/Au substrate or at the STM Au-tip, the magnetizations of the Co layer had been oriented in the opposite direction in order to preserve the symmetry of the *IV*-curves. This is the first time that the CISS effect is demonstrated for a tunneling junction without a direct interface between the helical molecules and the magnetic substrate. Our results can be explained by a spin-polarized or spin-selective interface effect, induced and defined by the helicity and electric dipole orientation of the molecule at the interface. In this sense, the helical molecule does not act as a simple spin-filter or spin-polarizer and the CISS effect is not limited to spinterfaces.

## Abstract 2

GMR (a) and TMR (b) device structures realized by using helical molecules adsorbed on the substrate and STM-tip, respectively.

## Graphical abstract

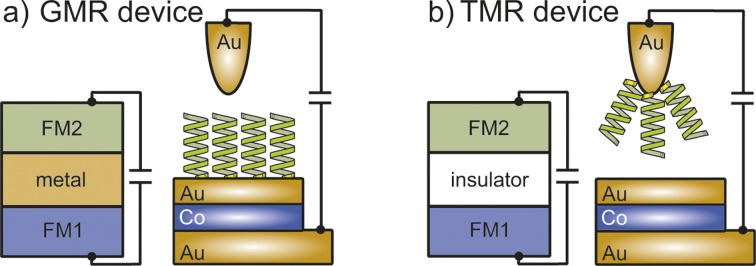

## Introduction

The chirality-induced-spin-selectivity (CISS) effect found in organic molecules has been intensively discussed for many years now and demonstrated in various experiments, partly opening new fields in physics and chemistry.^1–8^ Thereby, often electronic transport measurements with helical systems on magnetic substrates were performed showing a robust magnetoresistance (MR) effect in 2-terminal spin-valve measurements.^9^

Despite the numerous experiments that reveal the CISS effect, there is still no comprehensive explanation for the magnitude of the effect or even the exact mode of operation, *i.e.* if a chiral system acts as a spin-filter or as a spin-polarizer. Recently, it was shown that the CISS effect is even present in molecules without being adsorbed on a solid state surface.^8^ Among others, models on spin accumulation at interfaces,^10,11^ transfer of orbital angular momentum^12^ and intrinsic spin–orbit coupling^13^ including various boosting mechanisms, *e.g.* spin–phonon coupling and electron–chiral vibron coupling^14,15^ are currently discussed.

From a fundamental point of view, a chiral system, if acting as a spin-filter and adsorbed on a magnetic substrate, violates Onsager's reciprocity relation at least in the linear regime.^16^ A way out of this dilemma is to introduce magnetization dependent electrostatic barriers at the hybrid interface.^17^ Thereby, the molecule is assumed to be spin selective with respect to the chirality and direction of motion of the electrons, and the selectivity does not depend on the length of the molecule.^16^ Therefore, in conjunction with a magnetic substrate, the MR curves are expected to be asymmetric with respect to the bias voltage for a fixed magnetization direction and inverted for the other direction of magnetization. This approach oversimplifies the picture disregarding that dissipation, multi channels, and substrate magnetization affect the results. In fact, many of the measured *IV*-curves are point-symmetric for each of the magnetization directions,^18,19^ showing that non-linear effects play a role. Moreover, the CISS effect was shown to be dependent on the lengths of the molecule,^5,20^ which is in the first approximation not expected for the spin-filter model, where the transmission and reflection of the electrons are a result of the spin state when entering the molecule.^16^

Other models rely on a rather intramolecular mechanism, where the interplay of chirality and spin–orbit interaction leads to spin polarization. The orthogonal part of the momentum acts on the gradient of the electric potential like an effective magnetic field that aligns the spins of the itinerant electrons within the molecular system.^21^ The model agrees with the experimental finding that the degree of spin polarization increases with the length of the molecule.^5,19,20^ Although theory can qualitatively explain the spin polarization within chiral molecules, the magnitude of the observed effect still remains elusive. Therefore, the role of electron correlations as well as the interaction with other quasiparticles, *e.g.* phonons, is currently investigated.^14,22^ The latter can at least plausibly explain the observed temperature dependence of the CISS effect.^23^

The spin–orbit coupling in hydrocarbons and peptides is *per se* too small in order to quantitatively explain the CISS effect; therefore, the importance of substrates and interfaces from heavy elements was pointed out in the past. Adsorption of chiral molecules on surfaces comes along with the so called magnetism induced by proximity of adsorbed chiral molecule (MIPAC) effect.^24–28^ In fact, the above mentioned model of an electrostatically generated MR in a chiral spin-filter in contact with a ferromagnet also highlights the importance of interfaces.^17^ Among others, the orbital polarization effect (OPE) is still under debate, where the chiral system is in (ohmic) contact with a strong spin–orbit coupled system.^12^ Thereby, the electron is mainly taking up orbital angular momentum within the helical system, which is then converted into spin polarization upon scattering into the heavy element substrate. Another approach, which also relies on the interaction of molecules with ferromagnetic surfaces, deals with spinterface effects. This model can quantitatively explain the size of the spin polarization as well as the experimentally measured *IV*-curves across the magnetic heterojunction.^11,29^

Although the helicity of the molecules determines the spin orientation, *e.g.* probed by 2-terminal spin-valve measurements, there are a few experimental reports which state that the MR effect also alters if only the intramolecular electric dipole is reversed, *e.g.* DNA and polypeptides, while the helicity remains the same.^30,31^ Apparently, the electrostatic field either at the interface or along the molecules, like in polypeptides, plays an important role in the CISS effect.

**Fig. 1:**
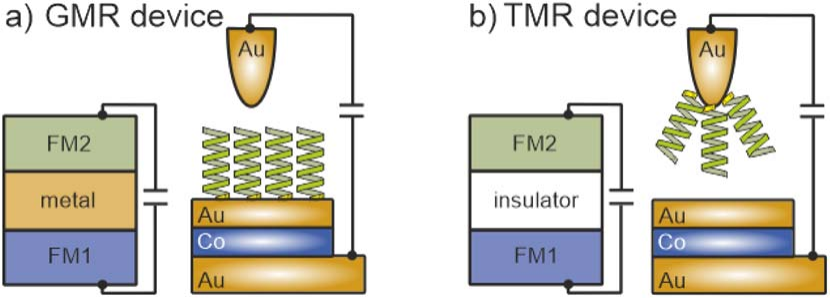
(a) GMR device structure: in a conventional GMR device the two ferromagnets (FM1 and FM2) are separated by a non-magnetic metallic layer. The hybrid version of this can be realized by adsorbing helical molecules on a Au/Co/Au substrate. (b) TMR device structure: FM1 and FM2 are separated by an insulating film. For the hybrid version this is realized by a tunneling gap in between the Au-tip with chemisorbed helical molecules and the Au/Co/Au substrate. For more details see the main text.

We have recently shown that STM not only can resolve the molecular structure on the surface, but also allows the measurement of the spin dependent MR.^32^ Thereby, the fixed layer is represented by the helical molecule, while the free layer is still a commonly used magnetic thin film of Co. A simplified model of this setup in comparison to a conventional giant magneto resistance (GMR) device is depicted in Fig. 1a, where the molecular layer on the Au surface results in a ferromagnetic fixed state (FM2) that can be probed by the magnetization of a Co-layer (FM1), which is freely switchable *via* an external magnetic field. The use of an STM setup not only allows for a bi-directional 2-terminal measurement, but also allows us to study the effect of external electric fields while choosing different set-points.

In this work, we have investigated spin-dependent transport through 16-mer polyalanine L-PA molecules using the spin-valve effect realized with an STM. The molecules were chemisorbed either directly onto a magnetic substrate or on the Au tip, *i.e.* in the latter case there is a tunneling junction for the spin-polarized electrons involved. This approach allows us to also realize, besides a hybrid GMR (*cf.*Fig. 1a), a TMR (tunneling MR) configuration, with helical molecules on one side and the FM Co freely switchable layer on the other side of the tunneling gap, as shown in Fig. 1b. For both scenarios we have found a spin polarization; however, in order to preserve the symmetry of the *IV* curves, the magnetization direction of the magnetic Co layer had to be oriented in the opposite direction for GMR and TMR configurations, and thus it is apparent that the spin polarization is directly related to the orientation of the electric dipole rather than the helicity of the molecule. The symmetry of the *IV* curves for both configurations can only be explained by the induction of spin-polarized electrons at the interfaces between Au and the chiral molecules.

## Experimental

### Materials and methods

We employ ambient scanning tunneling microscopy (STM) to characterize the structure and perform local scanning tunneling spectroscopy (STS) measurements. The STM-tip was made from a 0.25 mm Au wire. All measurements were performed at 300 K. In order to quantify the spin polarization of the transmitted electrons, *IV*-spectra were obtained at various set points (*V*_b_ = 0.5 V; *I*_t_ = 50 pA, 100 pA and 150 pA). All *IV* spectra shown are average values of at least 10 spectra.

**Fig. 2:**
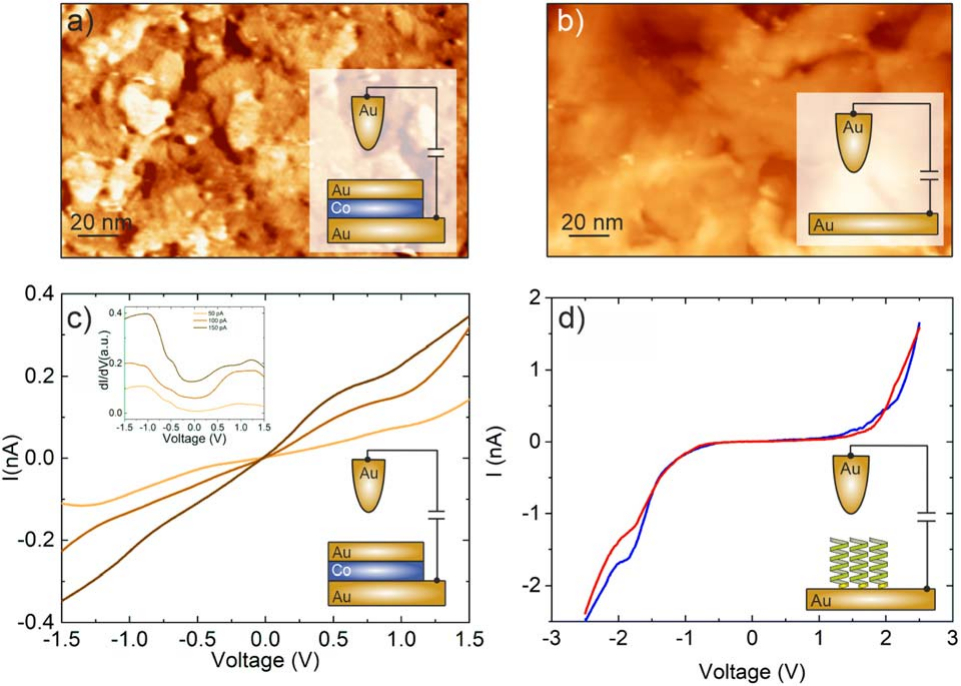
(a and b) STM images (125 × 200 nm^2^, *V* = 0.5 V, *I* = 150 pA, Au-tip) showing the topography of both parts of our samples (see the Materials and methods section for more details on the samples): (a) magnetic Au/Co/Au and (b) non-magnetic Au. The out-of-plane magnetization of the Co-film can be switched by using an external magnet (coercive field of 16 mT). The roughness (0.2 nm) and terrace sizes (20 nm) in both parts are comparable and suitable for STM. (c) *IV*-Curve obtained for the uncovered magnetic part at 3 different set points (50 (orange), 100 (brown) and 150 pA (darkbrown)). Inset: d*I*/d*V* curves showing the Au surface state at −0.6 eV. (d) *IV*-Curves after adsorption of 16-mer L-PA molecules on the non-magnetic part of the sample for an upward (red) and a downward magnetic field orientation (blue). The bias voltage was in all cases *V*_b_ = +0.5 V.

As substrates for the deposition of molecules we used specially designed MBE grown epitaxial magnetic nanostructures. The nanostructure consists of Al_2_O_3_/Pt/Au (20 nm) layers on which one half was further coated with Co (1.2 nm)/Au (5 nm) (hereafter denoted as Au/Co/Au). The Co layer reveals an out-of-plane magnetization with a coercive field of 16 mT, which can be easily switched by using an external magnet. The coercive field was determined by the polar magneto-optic Kerr effect and the corresponding magnetization curve with more details about the switching procedure can be found in ref. 32. The Au layers themselves on both halves of the sample revealed terrace sizes of around 20 nm, which are sufficient for our STM and STS measurements (*cf.*Fig. 2a and b). The adsorption of molecules on such surfaces divided into 2 parts allowed us to measure not only the transmission on the magnetic layer but also to obtain reliable reference measurements from the non-magnetic sample parts.

The L-PA molecules used in this study revealed the following sequence C[AAAAK]_3_ (16-mer), where C, A, and K represent cysteine (C), alanine (A), and lysine (K), respectively. The molecules were dissolved in absolute ethanol with a concentration of 0.3 mM. The adsorption of chiral 16-mer polyalanine (PA) molecules on the Au surfaces of both the STM tip and the substrates was performed by dipping and drop casting, respectively. The Au-tips were afterwards rinsed gently with absolute ethanol solvent several times in order to remove excess molecules from the tip surface.

## Results and discussion

### Reference measurements: role of the Co-layer and the MIPAC effect

Our spin-sensitive measurements are based on the spin-valve effect of transmitted electrons across a magnetic heterostructure, as discussed in the context of Fig. 1a.^32^ The use of a surface-sensitive scanning electron microscope is successful because the substrates used here are sufficiently smooth and suitable for the adsorption of molecules and local spectroscopy.^19^ In Fig. 2a and b we show the morphology of both the magnetic and non-magnetic parts of our sample, *i.e.* the Au/Co/Au and Au thin film only, respectively. The STM analysis revealed that on both parts the average terrace width is around 20 nm with an overall rms-roughness of about 0.2 nm.

To start with, we first show the tunneling behavior of a junction on the Au/Co/Au substrate without any PA molecules. The *IV*-spectra in Fig. 2c reveal a linear tunneling behavior at low bias voltages, which is indicative of a purely metallic system. Thereby, the tunneling current increases at smaller tip-surface distances, *i.e.* higher set point currents. In addition, at around −0.6 eV a characteristic shoulder can be recognized in the d*I*/d*V* spectra, shown as an inset, which can be attributed to the Shockley surface state of Au(111).^33^ Within the accuracy possible for the ambient STM measurements, we could not find any influence of the buried magnetic Co-layer on the STS spectra. Therefore, the topmost layer in the heterostructure resembles an Au(111) surface.

For the adsorption of helical systems, in particular polypeptides, it has been shown that magnetism in the substrate builds up during adsorption, which can switch the magnetization of other layers or trigger an enantiospecific adsorption on hard-magnetic surfaces.^26,28^ In order to see if this MIPAC effect plays a role in our spin-valve measurements, we adsorbed 16-mer L-PA molecules on the non-magnetic Au part of the sample and measured the *IV*-curve for two nominally different orientations of the externally approaching magnet potentially switching possible induced magnetization, like we do for the Au/Co/Au system. The *IV* curves are shown in panel d, mainly resembling the molecular energy gap of the PA molecules, similar to previous measurements with 36-mer.^34^ Such *IV*-curves for electrons transmitting through PA molecules were already reported before experimentally as well as theoretically.^35,36^ Most importantly, the spectra for both applied magnetic field directions are almost identical, showing no spin-valve effect without any additional magnetic layer. *i.e.* the CISS and MIPAC effects, if present, are strongly interlinked.

### Hybrid GMR configuration: L-PA molecules adsorbed on Au/Co/Au

In this section we will describe a study of the CISS effect by adsorbing the L-PA molecules on the surfaces of the Au/Co/Au samples, similar to previous experiments.^19^ The spin-polarized electrons are thereby probed using a 2-terminal spin-valve geometry. In view of the measurements in the following section, we will refer to this configuration as a hybrid GMR configuration.

**Fig. 3:**
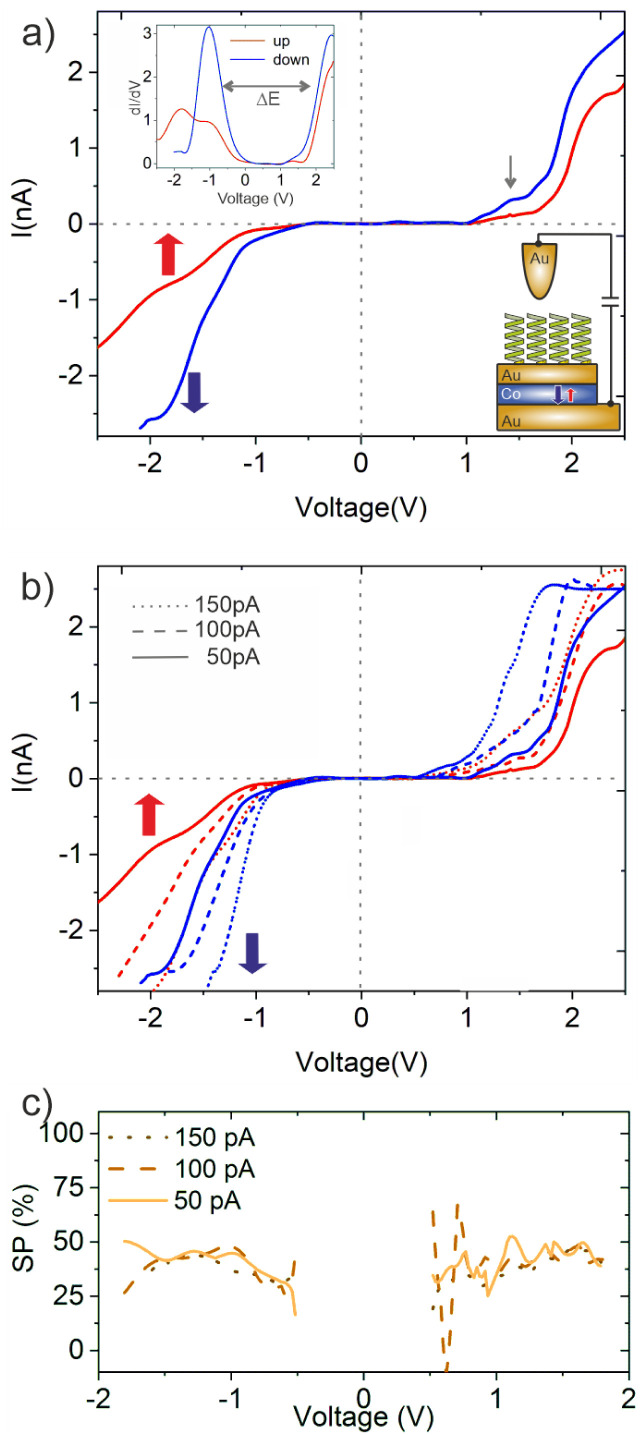
*IV*-Characteristics measured using a hybrid GMR configuration: (a) *IV* spectra (set point +0.5 V, 50 pA) obtained for 16-mer L-PA molecules adsorbed on a Au/Co/Au substrate for the two directions of the magnetization of the Co-layer, 
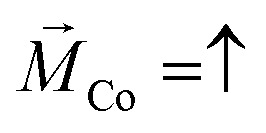
 up (red curve) and down 
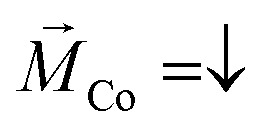
 (blue curve). The inset shows the d*I*/d*V* spectrum. The gray arrow denotes the spectral feature for the Au–S bond. (b) Similar *IV*-spectra, but obtained at different set point currents. With increasing set points, the absolute currents increase, while the SP, *i.e.* the relative difference of the currents for the two magnetization directions, remains roughly constant. All measurements were performed at 300 K with *V*_b_ = +0.5 V. (c) SP values obtained for L-PA chemisorbed on Au/Co/Au at different tunneling currents (set points).

Fig. 3a displays the *IV*-curves for 16-mer PA molecules adsorbed on the samples. In agreement with previous measurements, the adsorption of PA molecules on Au/Co/Au is mainly governed by the chemical bonding using the Au–S linkage, which is clearly characterized by the shoulder at around +1.4 eV.^34,37^ This Au–S bonding is formed by the chemical interaction between the Au(111) surface and thiol group connected to the N-terminal of the amine in the backbone of the PA molecules. The d*I*/d*V* curves, shown as an inset, contain peaks at around −1 V and +2 V associated with the highest occupied (HOMO) and lowest unoccupied molecular orbital (LUMO), respectively, revealing an electronic gap of around Δ*E* = 2.2–3.2 eV, which agrees well with calculations.^36^

In our previous measurements we demonstrated that the spin-polarization (SP) of the transmitted electrons through adsorbed PA molecules can be quantified by studying the *IV*-curves for different directions of the out-of-plane magnetization (up (red), 
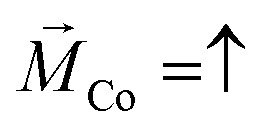
, and down (blue), 
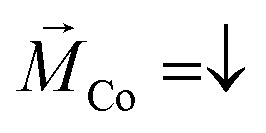
) of the Co-layer, 
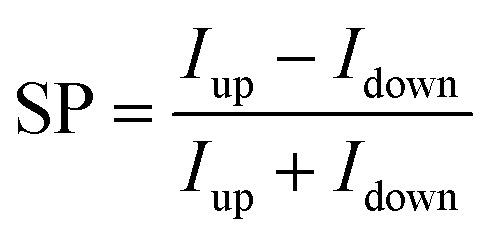
.^19,32^ Thereby, higher transmissions were found for L- and D-PA with downward and upward directions of the magnetization of the Co-layer, respectively.

The *IV*-curves shown in Fig. 3 follow this trend. For the 16-mer PA molecules probed at a set point of *V*_b_ = +0.5 eV and *I*_t_ = 150 pA we obtain a spin polarization value of around 48% measured at +1.4 eV (all SP values mentioned in the following section are collected at this voltage). The SP-values deduced for 26-mer and 31-mer L-PA molecules on similar Au/Co/Au systems were found to be 68% and 75%, respectively.^19,32,38^ The lower value for the 16-mer PA is apparently due to the shorter molecule length, and thus the overall SP measured is a molecular property and supports at this point that the helical system acts as a spin-polarizer.^7^ Moreover, the *IV* curves show a similar transmittance for positive and negative bias directions. In our previous study, we have seen that the SP-value is somewhat higher for electron hopping along the unoccupied states (positive bias voltages).^32^ This finding is not so apparent in this study using shorter molecules and might also be related to differences in the transport channels, *i.e.* non-coherent effects in longer molecules. Also, as we have shown before, the absolute values of the spin polarization depend on the ordering of the self-assembled PA layers,^19,32^ which in this case may be slightly worse. Nevertheless, these findings cannot be explained by a simple spin-filter model assumed for the helical system yielding different *IV*-curves,^17^ as sketched in Fig. 5a.

In addition, we systematically studied the electron transmissions for various set points of the tunneling current, *i.e.* tip-sample distances. The experiments were performed on the same structure and the data are shown in Fig. 3b. In accordance with higher current set points, the tunneling gap is smaller and the electron transmission is higher. However, this holds for both *IV*-curves measured for the upward and downward direction of the magnetization of the Co-film, so that the values of the spin polarization are more or less the same for a large bias range as shown in Fig. 3c. Apparently, the different electric fields realized by these different tunneling set points are not severely influencing the electronic structure of the molecule, *i.e.* the spin polarization.

### Hybrid TMR configuration: L-PA molecules attached to the Au-tip

So far, the molecules were chemisorbed on the magnetic heterostructure substrates. Since we are using an STM, working with a tunneling junction, we can easily realize a hybrid TMR configuration, *i.e.* using PA-functionalized Au-tips and bare magnetic substrates. In this case, assuming that the electron flow direction is from the tip towards the substrate and that the helical molecule is a spin-polarizer, spin-polarized electrons would tunnel across the tunneling barrier, while for the GMR setup (PA molecules adsorbed on a magnetic Au/Co/Au substrate) spin-unpolarized electrons are used in the tunneling process across the gap.^19^ We want to point out that for standard GMR and TMR configurations the conductance is expected to be the greatest for a parallel alignment of the spin majority in both subsystems.^39^

The STM-tip was functionalized with helical molecules *via* dipping the cut Au-wire into a 0.3 mM solution of 16-mer L-PA molecules. Thereby we assume that the molecules will bind with cysteine to the surface of the Au-tip. Compared to the adsorption of L-PA molecules on flat surfaces, we also expect a self-assembled monolayer structure, but less ordered.^34,40^

**Fig. 4:**
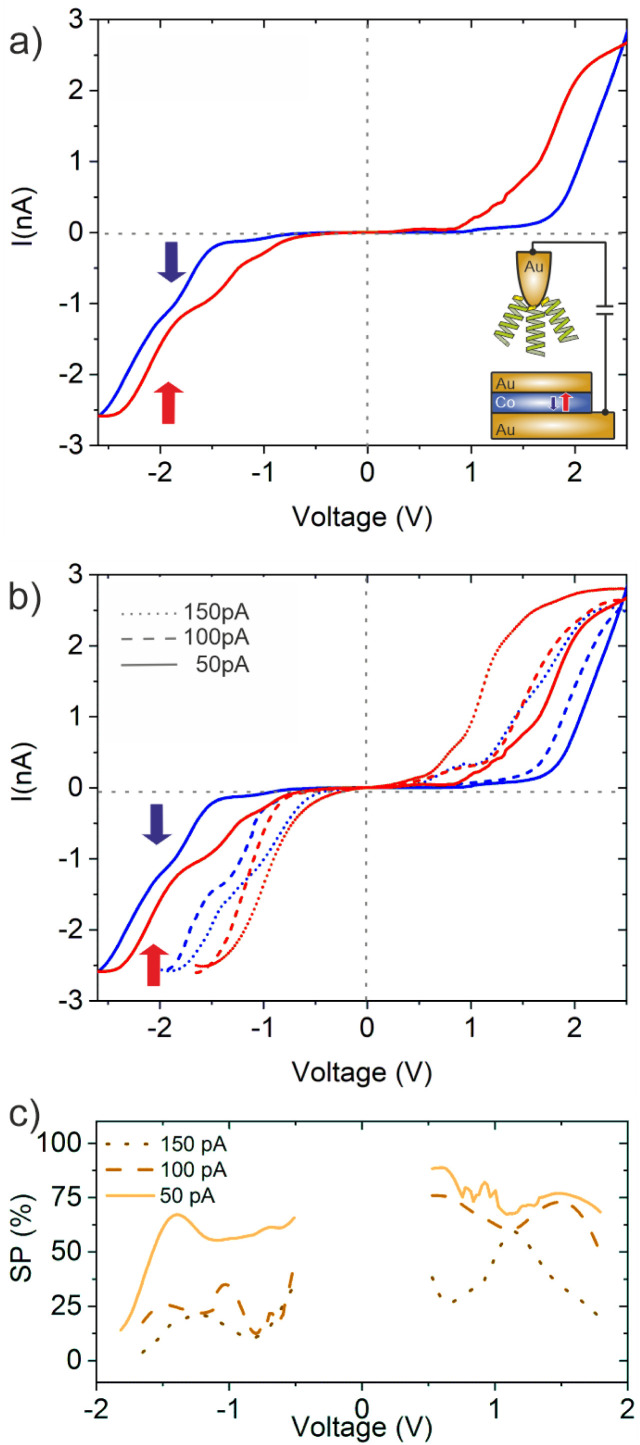
*IV*-Characteristics measured using a hybrid TMR configuration: (a) *IV* spectra (set point +0.5 V, 50 pA) obtained for 16-mer L-PA molecules adsorbed on the Au-tip for the two directions of the magnetization of the Co-layer, 
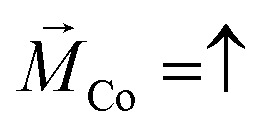
 up (red curve) and down 
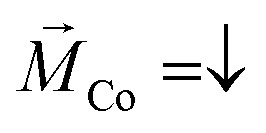
 (blue curve). The Au/Co/Au substrate was not covered by PA molecules. (b) Additional *IV* spectra with the same configuration as in panel (a), but with different current set points. (c) SP-values obtained for different set-points, *i.e.* electric fields across the junction. All measurements were performed at 300 K.

The *IV*-spectra, obtained for a set point *V*_b_ = +0.5 V and *I* = 50 pA are shown in Fig. 4a. Like for the case where the molecules were adsorbed on the Au/Co/Au surface (Fig. 3a), the *IV*-curves change for the two different directions of the magnetization of the Co layer. Again, the *IV*-curves are “point-symmetric”, *i.e.* a high conductance state is found for positive and negative bias voltages for the same direction of magnetization. Compared to the GMR scenario, the TMR configuration is also “CISS-active”, *i.e.* reveals a difference in the *IV*-curves for the two directions of magnetization. Although the CISS effect was found in the presence of tunneling junctions, *e.g.* MgO,^23^ this is the first time, to the best of our knowledge, that the CISS effect is demonstrated for a tunneling junction without a direct interface between the helical molecules and the magnetic substrate. While energy and spin of the electrons are conserved during the tunneling process outside the molecule across the additional tunneling barrier (air in this case), it is unlikely that the orbital angular momentum of the electrons in the helical system is conserved. Thus, our findings can hardly be explained with the orbital polarization effect (OPE) model mentioned above.^12^

However, in spite of all these similarities, there are two important differences compared to the case of GMR where the L-PA molecules are adsorbed on the sample surface: firstly, the spin-polarization is around 70%, *i.e.* almost doubled compared to that in the GMR scenario. Secondly, a high electron transmission is obtained in the case where the magnetization of the Co-layer is upwards, *i.e.* oppositely orientated to the GMR scenario shown in Fig. 3. We want to point out that the PA molecules in this study have the same length, linker group and helicity. The only parameter that has changed was the orientation of the electrostatic dipole and the sequence of molecules and the tunneling gap within the tunnel junction. Our results clearly show that the orientation of the dipole moment in PA molecules is crucial for the CISS effect. Similar observations regarding the importance of electric dipoles were made before,^30,31,41^ but are rarely considered in current models and theories. Moreover, the importance of the dipole alignment was also reported in the electron paramagnetic resonance experiments for helical molecular systems not adsorbed on surfaces but present in the solution.^8^

In order to further elucidate the effect of electric fields within the TMR configuration, we studied the dependence of the SP on different tunneling current set points. The results are summarized in Fig. 4c. For the highest set point (150 pA) we observe a spin polarization of only 30%, which is about half of the value which we found under the same conditions of molecules adsorbed on the Au/Co/Au surface (*cf.*Fig. 3b). The variation of the SP for molecules attached to the Au-tip is much stronger, showing an amplification and quenching for low and high electric fields across the junction, respectively. Thereby, the impact of the externally induced field, set by the bias voltage and the set point, does not depend on the sign of the applied voltage. The same also holds for the GMR configuration, although the variation of the SP values is much smaller. Therefore, the externally applied electric field is still smaller compared to the field induced by the electric dipole of the L-PA, which points for the 16-mer molecule always towards the cysteine, *i.e.* the surface to which it binds.

The electric field of the dipole can be rationalized as follows. For our 16-mer PA molecule, the dipole moment amounts to around 57 Debye. Neglecting screening effects by the environment and using a relative dielectric constant of *ε*_R_ = 2 for polyalanine,^42^ the electric field within the helical backbone structure is of the order of 10^8^ V cm^−1^, *i.e.* one to two orders of magnitude higher compared to the electric field applied across the entire junction. Therefore, the external field cannot overcompensate for the dipolar electric field of the PA molecule, but it seems to be able to alter the molecular configuration at the tip. One should also keep in mind that the electric field between the STM tip and substrate is inhomogeneous and much higher around the apex of the tip. In particular, the molecular orbital structure can be shifted by the Stark effect, as pointed out in transport experiments and seen in simulations.^35,36^ Based on this background, the strong changes in SP values for the configuration, where the molecules are at the tip, can be overshadowed by variations in orbital energies.

Although the variations of the absolute SP values for the TMR configuration can be rationalized by the externally applied field, our finding of a reversed magnetization of the Co layer for a high transmission state compared to the GMR configuration must have a molecular origin. In previous experiments, the orientation of the electric dipole moment of the PA molecules was altered with respect to the surface by using linker groups either at the C- or N-terminus, and it was found that the reorientation of the electric dipole also reverses the magnetic properties of the molecule/substrate system.^31,43^ Apparently, for the hybrid systems, this is an interface effect which in our case can also explain the reversal of the magnetization direction in the experiments with the molecule functionalized STM tip, *i.e.* the TMR configuration. At first sight, this finding seems to be contradictory to the CISS study performed on helical molecules without any substrates.^8^ However, in that case the helical systems were modified by donor and acceptor end groups.

In our case, direct tunneling of the spin-polarized electrons from the Au-tip into the magnetic substrate is unlikely, because the 16-mer molecule is already 2.5 nm in length. Since the currents for both the GMR and TMR configurations, as shown in Fig. 1, are comparable, we conclude that the spin-polarized electrons at the interface are transmitted through the molecules where they then tunnel into the substrate. Indeed, in our break-junction transport measurements we found a comparably high conductivity for the polypeptides, reflecting a low tunneling barrier for the electrons propagating along the helix, which decreases further for fields exceeding 5 × 10^5^ V cm^−1^.^35^ This quasi-ballistic behavior together with a small SOC within the molecules allows for an efficient transmittance of both spin-polarized and also spin-non-polarized electrons between the metallic contacts including a tunneling across the interface.

**Fig. 5:**
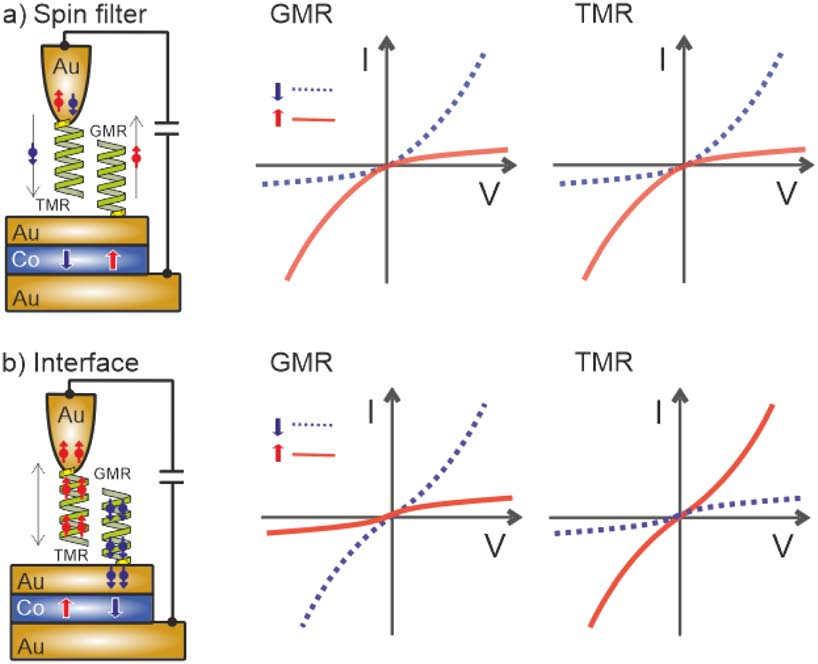
(a) Spin filter model: the spin selectivity of the molecule depends on its helicity and polarity, but not on its length. However, the direction of the current determines the selectivity of the molecules, *i.e.* the *IV*-curves reveal a diode-like behavior for each Co-film magnetization.^17^ Since the helicity of the molecule for the GMR and TMR configurations is the same, both configurations reveal similar *IV*-curves. (b) Interface model: the spin polarization at the interface is determined by the helicity and the dipole moment of the molecule. For the GMR and TMR configurations opposite spin polarizations are induced, and thus opposite Co-film magnetizations are needed in order to obtain identical curves. The transport of the spin-polarized electrons does not depend on the polarity of the bias voltage.

Assuming that the helical molecule itself acts as a simple spin-filter or spin-polarizer, as sketched in Fig. 5a, the expected d*IV* curves are non-symmetric upon reversing the Co-layer magnetization and voltage and thus contradict with our experimental results. In contrast, assuming that the spin polarization at the interface between the molecule and Au-substrate is determined by the helicity and the dipole moment of the molecules (see Fig. 5b), opposite spin polarizations are induced for the GMR and TMR configurations, and thus opposite Co-film magnetizations are needed in order to obtain identical curves. Moreover, the electronic transport of the spin-polarized electrons does not depend on the polarity of the bias voltage. Our results show clearly that a FM/molecule interface, as required for the spinterface effect, seems to play only a minor role in our experiments.

## Conclusions

In summary, we have realized hybrid GMR and TMR configurations with L-PA molecules chemisorbed either directly on the magnetic substrate or on the Au-tip, respectively. For both scenarios spin-polarized transport was found and quantified by a 2-terminal spin-valve effect using an STM. However, the direction of the magnetization of the Co-layer for the respective high/low resistance states in each case was found to be opposite for the GMR and TMR configurations and correlated directly with the orientation of the electric dipole of the molecule. The importance of electric fields at the interface is in line with investigations about the coupling of the L-PA molecules to the Au surface. The SP value for chemisorbed L-PA molecules is around 5% higher compared to physisorbed molecules.^19^ Generally, a strong symmetry breaking of the environment of the electrons when passing through the interface from a helical or chiral molecule into a cubic crystal, like Au, might be crucial for creating an electric dipole moment at this interface.

This is the first time, to the best of our knowledge, that the CISS effect is demonstrated for a tunneling junction without a direct interface between the helical molecules and the ferromagnetic substrate, as required for a spinterface. While the absolute values for the SP can be changed within certain limits by the externally applied field in the case where the molecules are adsorbed on the tip, the SP value is rather constant in the case of the 2D film on the substrate. The CISS effect seen for all configurations does not strongly depend on the sign of the bias voltage, *i.e.* the direction of the current. This suggests that the CISS effect is rather an interface effect and closely entangled with the MIPAC effect while being first order independent of including a spinterface within the hybrid spin valve structure. The helicity and molecular dipole orientation are important and determine the sign and magnitude of the spin polarization at the interface. Therefore, the molecule itself cannot be disentangled from the surface and the contacts, so the full system should be considered in any CISS model. In a wider context, such interfaces which introduce new states can also apparently be generated by chemical means, *i.e.* donor and acceptor sites at the end of chiral molecules.^8^ Spin–orbit coupling of chiral molecules adsorbed on surfaces is essential to break the symmetry and induce CISS. However, weak SOC that exists in chiral organic molecules seems to be sufficient to ensure a spin-conserving transport through the molecules. Regarding the spin polarization at the interface, there are different scenarios conceivable: within the framework of the Bychko–Rashba effect, Zeeman-like states for electrons in surface states can be achieved by a continuous reduction of the symmetry.^44^ In addition, the formation of spin-dependent barriers or charge transfer at molecular interfaces also cannot be ruled out.^17,45^ Which effect or which combination of effects is important needs to be clarified in future studies.

## Author contributions

T. N·H·N. performed the measurements and analyzed the data. T. N. H. N., G. S., O.·H., Y.·P., and L. T. B. participated in data analysis and writing the manuscript. L. T. B. provided magnetic nanostructures and their magnetic characterization. C. T. coordinated the experiments, analyzed the data and wrote the manuscript.

## Conflicts of interest

The authors declare no competing financial interest.

## Data Availability

The data are available on request from the authors.

## References

[cit1] Xie Z., Markus T. Z., Cohen S. R., Vager Z., Gutierrez R., Naaman R. (2011). Nano Lett..

[cit2] Naaman R., Paltiel Y., Waldeck D. H. (2019). Nat. Rev. Chem.

[cit3] Naaman R., Paltiel Y., Waldeck D. H. (2020). J. Phys. Chem. Lett..

[cit4] Ghosh S., Mishra S., Avigad E., Bloom B. P., Baczewski L. T., Yochelis S., Paltiel Y., Naaman R., Waldeck D. H. (2020). J. Phys. Chem. Lett..

[cit5] Waldeck D. H., Naaman R., Paltiel Y. (2021). APL Mater..

[cit6] Yang S.-H., Naaman R., Paltiel Y., Parkin S. S. P. (2021). Nat. Rev. Phys..

[cit7] Aiello C. D., Abendroth J. M., Abbas M., Afanasev A., Agarwal S., Banerjee A. S., Beratan D. N., Belling J. N., Berche B., Botana A., Caram J. R., Celardo G. L., Cuniberti G., Garcia-Etxarri A., Dianat A., Diez-Perez I., Guo Y., Gutierrez R., Herrmann C., Hihath J., Kale S., Kurian P., Lai Y.-C., Liu T., Lopez A., Medina E., Mujica V., Naaman R., Noormandipour M., Palma J. L., Paltiel Y., Petuskey W., Ribeiro-Silva J. C., Saenz J. J., Santos E. J. G., Solyanik-Gorgone M., Sorger V. J., Stemer D. M., Ugalde J. M., Valdes-Curiel A., Varela S., Waldeck D. H., Wasielewski M. R., Weiss P. S., Zacharias H., Wang Q. H. (2022). ACS Nano.

[cit8] Eckvahl H. J., Tcyrulnikov N. A., Chiesa A., Bradley J. M., Young R. M., Carretta S., Krzyaniak M. D., Wasielewski M. R. (2023). Science.

[cit9] Huizi-Rayo U., Gutierrez J., Seco J. M., Mujica V., Diez-Perez I., Ugalde J. M., Tercjak A., Cepeda J., San Sebastian E. (2020). Nano Lett..

[cit10] Naskar S., Mujica V., Herrmann C. (2023). J. Phys. Chem. Lett..

[cit11] Alwan S., Sharoni A., Dubi Y. (2024). J. Phys. Chem. C.

[cit12] Liu Y., Xiao J., Koo J., Yan B. (2021). Nat. Mater..

[cit13] Gutierrez R., Díaz E., Naaman R., Cuniberti G. (2012). Phys. Rev. B.

[cit14] Fransson J. (2020). Phys. Rev. B.

[cit15] Fransson J. (2023). Phys. Rev. Res..

[cit16] Yang X., van der Wal C. H., van Wees B. J. (2019). Phys. Rev. B.

[cit17] Tirion S. H., van Wees B. J. (2024). ACS Nano.

[cit18] Mondal A. K., Preuss M. D., Sleczkowski M. L., Das T. K., Vantomme G., Meijer E. W., Naaman R. (2021). J. Am. Chem. Soc..

[cit19] Ha Nguyen T. N., Paltiel Y., Baczewski L. T., Tegenkamp C. (2023). ACS Appl. Mater. Interfaces.

[cit20] Kettner M., Göhler B., Zacharias H., Mishra D., Kiran V., Naaman R., Waldeck D. H., Sek S., Pawlowski J., Juhaniewicz J. (2015). J. Phys. Chem. C.

[cit21] Naaman R., Waldeck D. H. (2015). Annu. Rev. Phys. Chem..

[cit22] Fransson J. (2019). J. Phys. Chem. Lett..

[cit23] Das T. K., Tassinari F., Naaman R., Fransson J. (2022). J. Phys. Chem. C.

[cit24] Sukenik N., Tassinari F., Yochelis S., Millo O., Baczewski L. T., Paltiel Y. (2020). Molecules.

[cit25] Meirzada I., Sukenik N., Haim G., Yochelis S., Baczewski L. T., Paltiel Y., Bar-Gill N. (2021). ACS Nano.

[cit26] Banerjee-Ghosh K., Ben Dor O., Tassinari F., Capua E., Yochelis S., Capua A., Yang S.-H., Parkin S. S. P., Sarkar S., Kronik L., Baczewski L. T., Naaman R., Paltiel Y. (2018). Science.

[cit27] Tassinari F., Steidel J., Paltiel S., Fontanesi C., Lahav M., Paltiel Y., Naaman R. (2019). Chem. Sci..

[cit28] Ben Dor O., Yochelis S., Radko A., Vankayala K., Capua E., Capua A., Yang S.-H., Baczewski L. T., Parkin S. S. P., Naaman R., Paltiel Y. (2017). Nat. Commun..

[cit29] Alwan S., Dubi Y. (2021). J. Am. Chem. Soc..

[cit30] Eckshtain-Levi M., Capua E., Refaely-Abramson S., Sarkar S., Gavrilov Y., Mathew S. P., Paltiel Y., Levy Y., Kronik L., Naaman R. (2016). Nat. Commun..

[cit31] Clever C., Wierzbinski E., Bloom B. P., Lu Y., Grimm H. M., Rao S. R., Horne W. S., Waldeck D. H. (2022). Isr. J. Chem..

[cit32] Nguyen T. N. H., Rasabathina L., Hellwig O., Sharma A., Salvan G., Yochelis S., Paltiel Y., Baczewski L. T., Tegenkamp C. (2022). ACS Appl. Mater. Interfaces.

[cit33] Chen W., Madhavan V., Jamneala T., Crommie M. F. (1998). Phys. Rev. Lett..

[cit34] Ha N. T. N., Sharma A., Slawig D., Yochelis S., Paltiel Y., Zahn D. R. T., Salvan G., Tegenkamp C. (2020). J. Phys. Chem. C.

[cit35] Slawig D., Nguyen T. N. H., Yochelis S., Paltiel Y., Tegenkamp C. (2020). Phys. Rev. B.

[cit36] Cristancho D., Seminario J. M. (2010). J. Chem. Phys..

[cit37] Sharma A., Matthes P., Soldatov I., Arekapudi S. S. P. K., Böhm B., Lindner M., Selyshchev O., Thi Ngoc Ha N., Mehring M., Tegenkamp C., Schulz S. E., Zahn D. R. T., Paltiel Y., Hellwig O., Salvan G. (2020). J. Mater. Chem. C.

[cit38] Nguyen T. N. H., Sharma A., Slawig D., Yochelis S., Paltiel Y., Zahn D. R. T., Salvan G., Tegenkamp C. (2020). J. Phys. Chem. C.

[cit39] Julliere M. (1975). Phys. Lett. A.

[cit40] Nguyen T. N. H., Solonenko D., Selyshchev O., Vogt P., Zahn D. R. T., Yochelis S., Paltiel Y., Tegenkamp C. (2019). J. Phys. Chem. C.

[cit41] Carmeli I., Skakalova V., Naaman R., Vager Z. (2002). Angew. Chem., Int. Ed..

[cit42] Calvo F., Dugourd P. (2008). Biophys. J..

[cit43] Carmeli I., Naaman R., Leitus G., Reich S., Vager Z. (2003). Isr. J. Chem..

[cit44] Nakajin K., Murakami S. (2015). Phys. Rev. B.

[cit45] Ma'Mari F. A., Moorsom T., Teobaldi G., Deacon W., Prokscha T., Luetkens H., Lee S., Sterbinsky G. E., Arena D. A., MacLaren D. A., Flokstra M., Ali M., Wheeler M. C., Burnell G., Hickey B. J., Cespedes O. (2015). Nature.

